# Tracing links between micronutrients and type 2 diabetes risk: the singular role of selenium

**DOI:** 10.3389/fendo.2024.1422796

**Published:** 2024-10-14

**Authors:** Wenxing Zeng, Shan Jiang, Dejun Cun, Feng Huang, Ziwei Jiang

**Affiliations:** ^1^ First Clinical Medical College of Guangzhou University of Chinese Medicine, Guangzhou, China; ^2^ Guangzhou University of Chinese Medicine Huizhou Hospital, Huizhou, China; ^3^ Department of Traumatology and Orthopedics, The First Affiliated Hospital of Guangzhou University of Chinese Medicine, Guangzhou, China

**Keywords:** selenium, type 2 diabetes, causality, Mendelian randomization, multivariate analysis, micronutrients

## Abstract

**Background:**

Type 2 diabetes (T2D) is a growing global health concern. While micronutrients are crucial for physiological functions and metabolic balance, their precise links to T2D are not fully understood.

**Methods:**

We investigated the causal relationships between 15 key micronutrients and T2D risk using both univariate and multivariate Mendelian randomization (MR) methods. Our analysis leveraged data from a large prospective cohort genome-wide association study (GWAS) on these micronutrients and T2D. We employed MR techniques such as inverse variance weighting (IVW), MR Egger, weighted median, and simple models. Multivariate analysis adjusted for diabetes-related factors like body mass index (BMI) and hypertension to assess the independent effects of micronutrients, particularly selenium, on T2D risk.

**Results:**

Selenium intake was associated with an increased risk of T2D, with an odds ratio (OR) of 1.045, a 95% confidence interval (CI) ranging from 1.009 to 1.082, and a *P*-value of 0.015. This association was consistent in multivariate analyses, suggesting an independent effect of selenium on T2D risk after adjusting for confounders.

**Conclusion:**

Our study presents novel evidence of a positive correlation between selenium intake and T2D risk, underscoring the importance of micronutrients in diabetes prevention and treatment strategies. Further research is necessary to confirm these findings and to clarify the specific biological mechanisms through which selenium influences diabetes risk.

## 1 Introduction

T2D is a global chronic metabolic disorder characterized by insulin resistance and relative insulin deficiency (1). According to the latest projections from the International Diabetes Federation, approximately 425 million adults worldwide currently suffer from diabetes, with T2D comprising the majority. By 2045, this number is expected to rise to 700 million, with an estimated 4.2 million adults aged 20-79 succumbing to diabetes, accounting for 11.3% of all deaths, predominantly in low- and middle-income countries (2). This increase correlates closely with rising obesity rates and lifestyle changes. Prolonged uncontrolled hyperglycemia can lead to serious complications such as cardiovascular disease, nephropathy, neuropathy, and retinopathy, imposing significant health burdens on patients and substantial economic pressures on healthcare systems (3).

While the genetic basis of T2D has been extensively studied, environmental factors, particularly micronutrient intake, appear equally critical in disease development. Micronutrients like calcium, magnesium, iron, and zinc play pivotal roles not only in maintaining metabolic health but also potentially in directly or indirectly regulating glucose metabolism through insulin secretion and action (4, 5). For instance, magnesium, as a cofactor for over 300 enzymes, plays a crucial role in glucose metabolism and insulin signal transduction (4). Despite widespread exploration of the relationship between micronutrients and T2D in epidemiological studies, existing evidence remains inconsistent due to potential confounding factors and reverse causation. For example, studies such as the Australian Longitudinal Study (6) and Nurses’ Health Study (7) have concluded that higher zinc intake, compared to the lowest quintile, correlates with lower T2D risk. However, in the Multi-Ethnic Study of Atherosclerosis, baseline dietary zinc or supplement use showed no association with T2D risk over a 10-year follow-up (8, 9).

MR emerges as a novel epidemiological tool to explore causal relationships within observational data (10). This method utilizes genetic variants as instrumental variables to help address confounding and reverse causation issues inherent in traditional observational studies (11). Our study aims to employ MR to comprehensively assess the potential causal relationships between 15 major micronutrients and T2D. By analyzing data from large prospective cohorts and GWAS, we will utilize MR to investigate the associations between genetic markers of these micronutrients and T2D risk. Additionally, we will employ multivariable analysis methods to control for other disease-related potential factors, thereby enhancing the accuracy of our causal inferences.

Our research not only promises new insights into the etiology of T2D but may also have profound implications for nutritional guidance, disease prevention, and public health policy formulation. By elucidating causal relationships between micronutrients and T2D, our study aims to better understand the complex pathogenesis of T2D and provide scientific foundations for future prevention and treatment strategies.

## 2 Materials and methods

### 2.1 Methods

**Figure 1:**
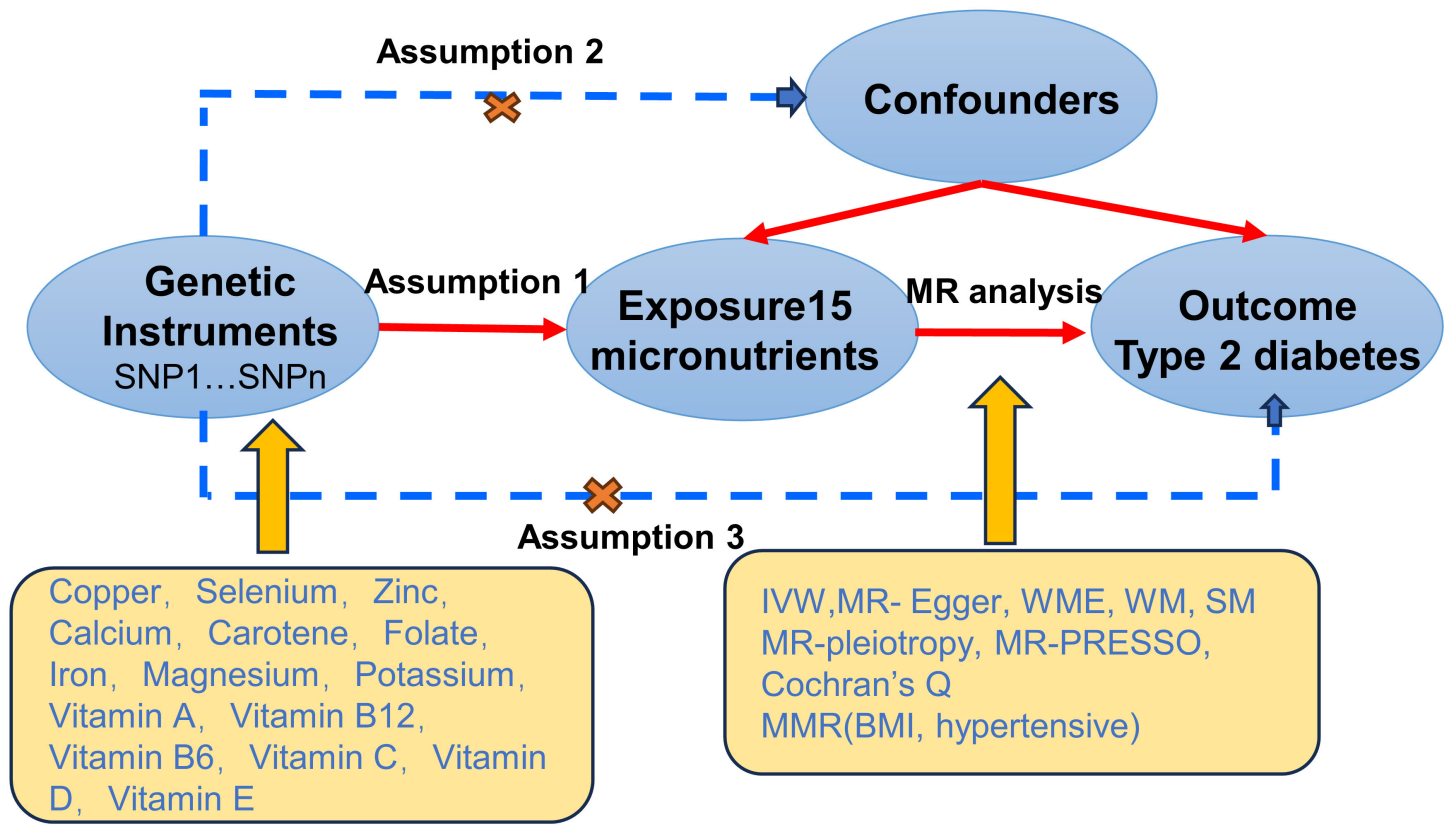
Mendelian Randomisation (MR) design flowchart.

This study utilizes MR to explore potential causal relationships between 15 micronutrients and T2D. Initially, genetic instrumental variables (SNPs) for these micronutrients are extracted from published GWAS. Subsequently, these instrumental variables are used in two-sample MR analyses to preliminarily screen for micronutrients significantly associated with T2D risk. Building on this, to control for the influence of other confounding factors on T2D, we incorporate BMI and hypertension—two closely related risk factors—into a multivariable MR analysis. This analysis aims to assess the independent association between selenium and other confounding factors with T2D risk. Additionally, MR design must meet three essential conditions (12): (A) Genetic variants chosen as instrumental variables (IVs) should be closely associated with each of the 15 micronutrients; (B) The genetic instruments should be unrelated to the outcome of type 2 diabetes and independent of potential confounding factors; (C) Genetic variants should be specifically associated with T2D through micronutrients rather than other pathways. **Figure 1** illustrates our workflow.

### 2.2 Data sources for GWAS

#### 2.2.1 Data sources for 15 micronutrients

Elsworth et al. (13) synthesized data from MRC-IEU, providing comprehensive GWAS data on micronutrients. The study included 64,979 individuals from European cohorts, covering 9,851,867 SNPs. This dataset encompasses 12 micronutrients: calcium, carotenoids, folate, iron, magnesium, potassium, vitamins A, B12, B6, C, D, and E. Additionally, GWAS data for copper, selenium, and zinc were sourced from Evans et al. (14), involving 2,603 individuals and 2,543,646 SNPs. All samples underwent rigorous quality control and are stored in IEU OpenGWAS (https://gwas.mrcieu.ac.uk); specific GWAS IDs are detailed in **Supplementary Table S1**.

#### 2.2.2 Outcome data and confounders data

Summary data on SNPs related to T2D are from the latest FinnGen (https://www.finngen.fi/en/access_results), version R10, under finngen_R10_T2D. This dataset aggregates GWAS results from 400,197 individuals (65,085 T2D cases and 335,112 controls) (15). T2D cases were diagnosed based on International Classification of Diseases (ICD)-8, ICD-9, and ICD-10 criteria by the Finnish Genetic Alliance. All statistical data used in our study are publicly accessible, thus ethical approval was not required. Detailed information on outcome data is provided in **Supplementary Table S2**.

For the multivariable MR study, BMI GWAS data encompassed 461,460 individuals of European descent, with 79,851,867 SNPs, sourced from IEU OpenGWAS under the ID ukb-b-19953. Hypertension GWAS data came from the Finnish database (finn-b-I9_HYPERTENSION), including 55,955 hypertension cases and 162,837 healthy controls, totaling 16,380,466 SNPs.

### 2.3 Selection of instrumental variables

In this study, SNPs with initial analysis p-values below the genome-wide significance threshold (5 × 10^-6) were chosen as IVs to ensure comprehensive results and enhance IV sensitivity. Subsequently, all IVs underwent linkage disequilibrium clumping (r^2 = 0.001; distance=10,000 kb) to minimize the influence of correlated SNPs. Furthermore, we utilized Phenoscanner (http://www.phenoscanner.medschl.cam.ac.uk/) to screen for potential pleiotropic effects and exclude SNPs associated with outcomes (see **Supplementary Table S3**). We calculated the F-statistic [R^2(N-2)/(1-R^2)] for each instrument to assess its strength, where R^2 represents the proportion of variance explained by genetic instruments and N is the effective sample size of the GWAS. SNPs with an F-statistic greater than 10 were selected for subsequent MR analysis, providing reliable estimates of genetic variation (16).

### 2.4 Mendelian randomization analysis

We conducted two-sample MR analyses to investigate the causal relationships between 15 micronutrients and T2D. The methods employed include the IVW approach (17, 18), weighted median (19), simple model, and MR Egger (20). The IVW method utilizes the variance of each IV to determine its weight, thereby mitigating the impact of instruments with larger variances on the results. This method assumes all IVs are valid and independent of the outcome, providing unbiased estimates of the causal effects of exposure on the outcome, thus serving as the primary test method. MR Egger combines traditional IV regression analysis with a method based on quantiles. It detects and corrects for potential issues with invalid instruments, such as measurement errors or correlations between IVs and confounding factors, making it a crucial tool for sensitivity analysis. The weighted median method reduces the influence of estimates with larger variances by assigning different weights based on their variances, aiming to provide a more robust causal estimate and minimize the impact of outliers or extreme values. It allows up to half of the SNPs to be invalid instruments or exhibit pleiotropy. Additionally, the weighted model and simple model serve as supplementary methods to IVW. Consistency among these five methods enhances the reliability of the results, ensuring all effect estimates (ORs) are in the same direction. Cochran’s Q test (21) evaluates heterogeneity among instruments. Furthermore, sensitivity analyses using MR pleiotropy residual sum and outlier (MR-PRESSO) (22) assess statistical significance and evidence of potential causal effects, excluding significance levels of *P* < 0.05. Leave-one-out sensitivity analysis (23) and MR Egger intercept can serve as supplementary indicators for sensitivity analysis, ensuring the reliability and robustness of our final results.

### 2.5 Multivariate Mendelian randomization analysis

As an extension of two-sample MR, multivariable MR estimates the combined causal effects of various risk factors on T2D susceptibility (24) by including all exposures in the same model. To demonstrate the independent direct effect of selenium as a trace element on T2D risk, controlling for BMI and hypertension, and confirming this effect is not mediated through other exposures, we selected significantly associated SNPs and integrated them with existing exposure IVs. After excluding these duplicate SNPs, we obtained the effects of each SNP and their corresponding standard errors from exposure and outcome. The core analytical method IVW was still employed to infer causal relationships in multivariable MR analysis.

## 3 Results

### 3.1 Selection of Ivs

**Supplementary Table S4** provides detailed information on SNPs associated with the 15 micronutrients, including β-values, standard errors, effect alleles, and other relevant data. The F-statistic values for the selected SNPs range from 20.87 to 84.68, indicating robust instrumental strength and supporting the absence of weak instrumental bias in our analyses.

### 3.2 Causal association between 15 micronutrients and T2D mellitus

**Figure 2:**
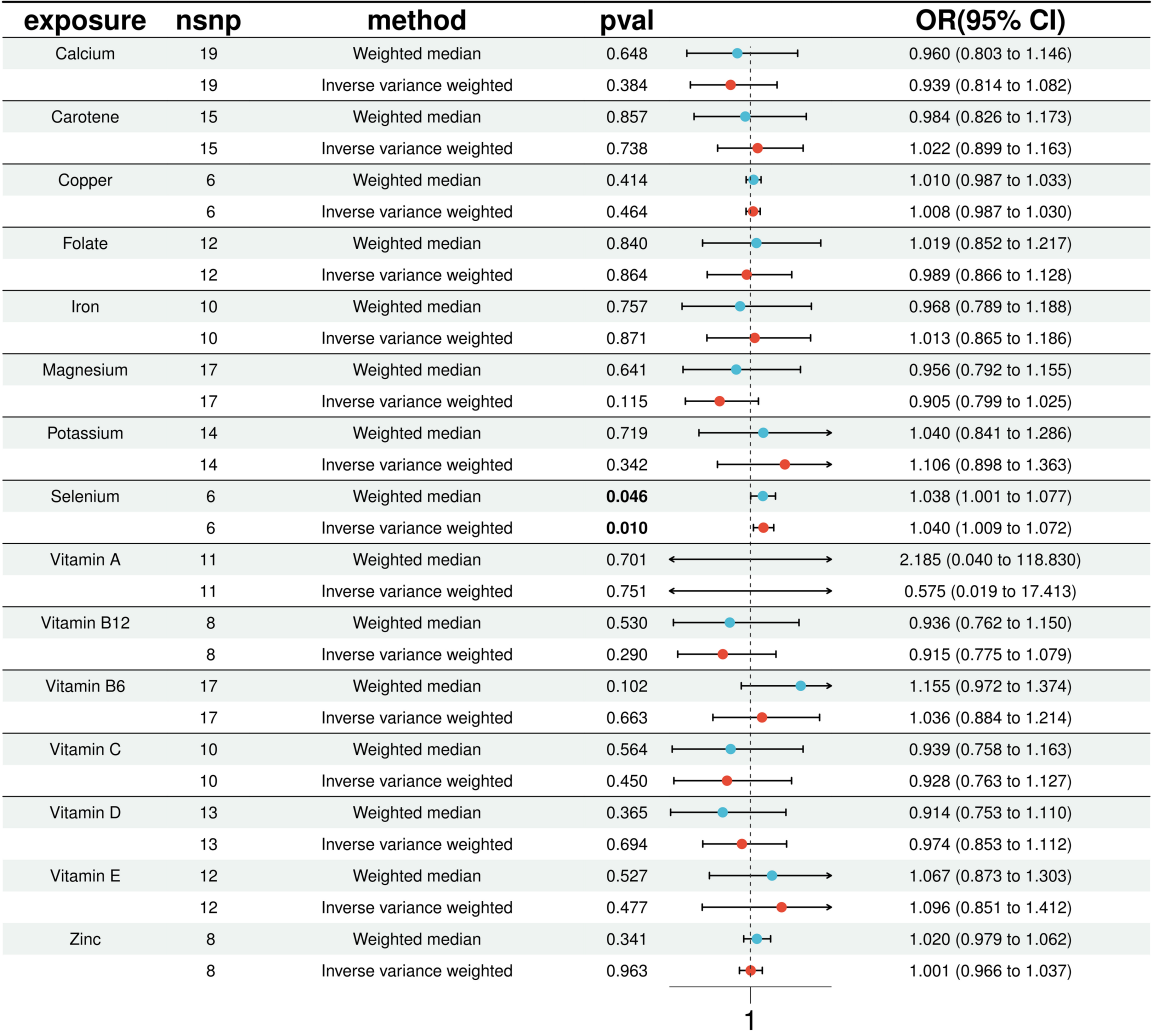
Results of 15 micronutrients with T2D MR analysis (IVW & WME).

**Figure 3:**
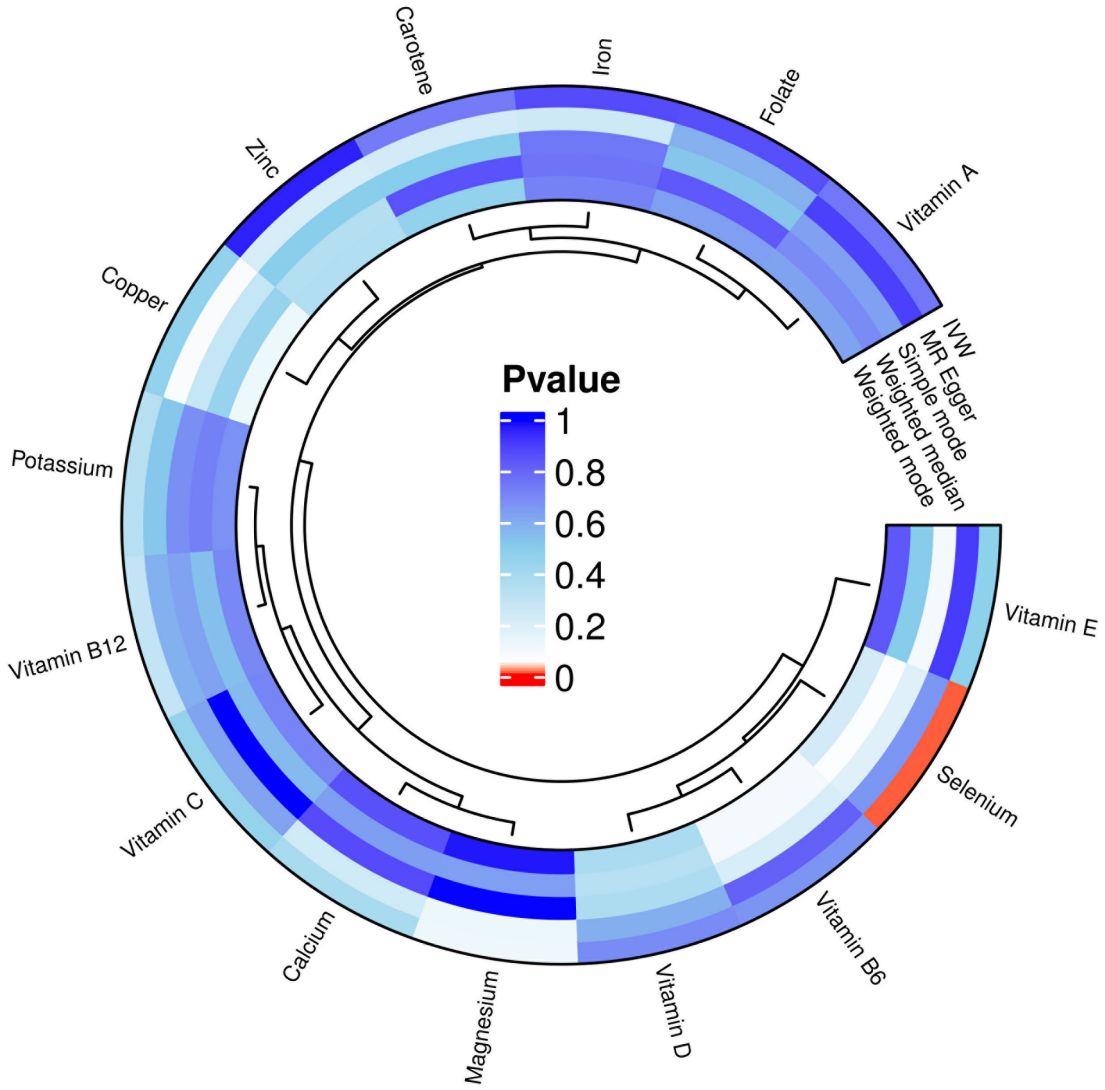
Positive results in 15 micronutrients.

In our MR analysis, we assessed the potential causal relationships between 15 micronutrients and the risk of T2D using multiple MR methods, including IVW, MR Egger, weighted median, simple model, and weighted mode. For micronutrients such as calcium, iron, folate, and vitamin B6, the *P*-values exceeded 0.05, indicating no significant associations with T2D risk. **Figure 2** provides comprehensive results for all 15 micronutrients. Notably, selenium exhibited a significant association, detailed in **Figure 3**.

**Figure 4:**
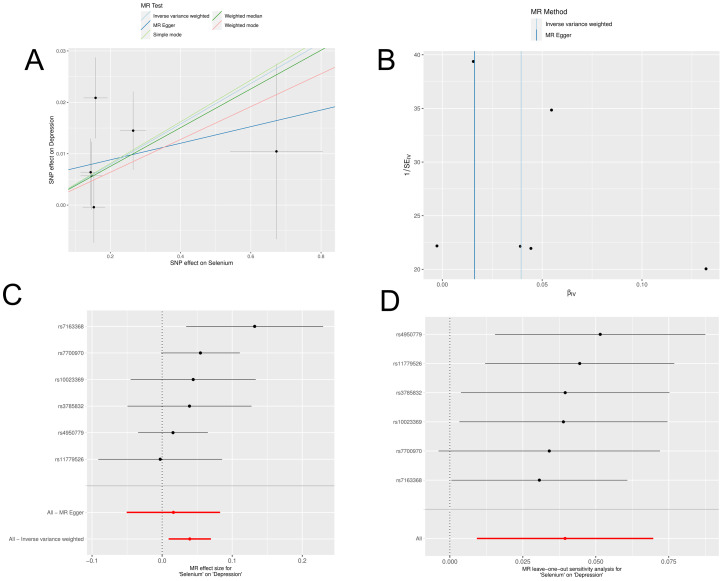
Results of MR analyses of selenium versus T2D, including **(A)** scatter plots, **(B)** forest plots, **(C)** funnel plots, **(D)** leave-one-out method.

**Figure 5:**
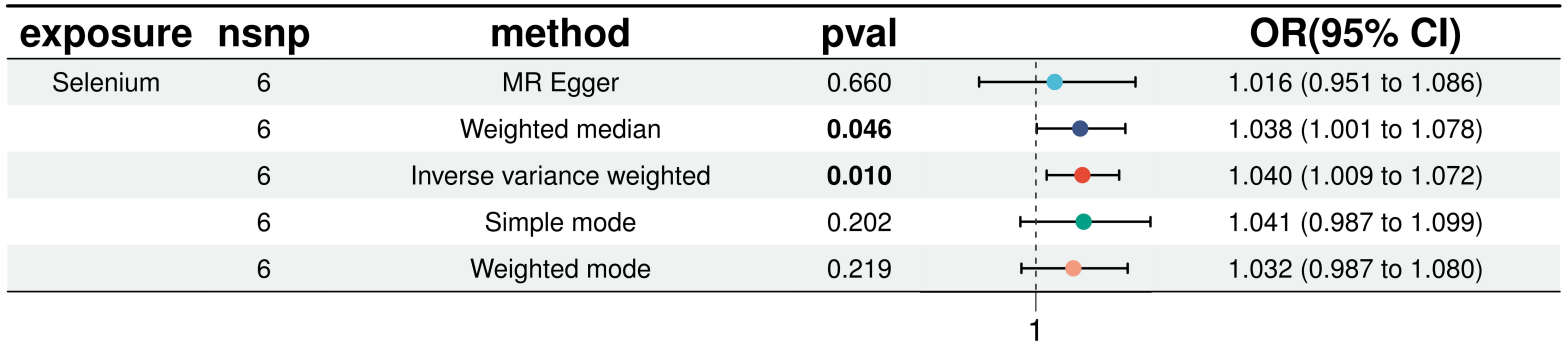
Results of five MR analyses between elemental selenium and T2D.

In a detailed two-sample MR analysis, selenium significantly increased T2D risk by 45% per standard deviation increase in its levels (95% CI: 1.009 to 1.082, *P* = 0.015). Results from MR Egger, WME, IVW, SM, and WM methods were as follows: MR Egger: OR = 1.016 (95% CI: 0.951-1.086, *P* = 0.660); WME: OR = 1.038 (95% CI: 1.001-1.078, *P* = 0.046); IVW: OR = 1.040 (95% CI: 1.009-1.072, *P* = 0.010); SM: OR = 1.041 (95% CI: 0.987-1.099, *P* = 0.202); WM: OR = 1.032 (95% CI: 0.987-1.080, *P* = 0.219). All methods consistently indicated ORs above 1, supporting a positive association between selenium and T2D risk, detailed in **Figure 4**. While copper showed a positive trend toward association with T2D risk, it did not reach statistical significance (*P* = 0.922). **Figure 5** displays various MR plots for selenium, including scatterplot, forest plot, funnel plot, and leave-one-out method. Our pleiotropy analyses, primarily using MR-pleiotropy, indicated no significant bias (*P* > 0.05). The MR-Egger intercept, detecting horizontal pleiotropy, also supported the absence of such bias with all p-values above 0.05. Furthermore, MR-PRESSO analysis, assessing heterogeneity, detected no outliers, indicating robustness. Leave-one-out sensitivity analysis confirmed that no individual SNP disproportionately influenced the overall effect. Additionally, Cochran’s Q test (*P* = 0.36) showed no significant heterogeneity, confirming the robustness of our findings and the consistency of complementary analyses.

### 3.3 Multivariate Mendelian randomization analysis

**Figure 6:**
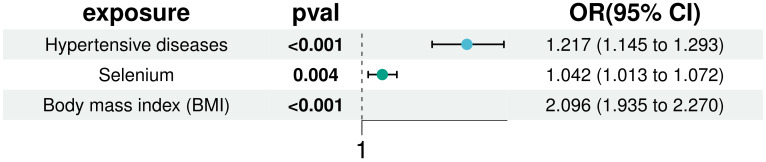
Multivariate MR analysis to correct for the confounding effects of BMI and hypertension on the effects of selenium and T2D.

In our multivariate analyses, we rigorously adjusted for BMI and hypertension to assess the independent association of selenium with T2D risk. The positive correlation between selenium and T2D risk remained statistically significant even after controlling for these established diabetes risk factors (IVW: OR = 1.042, 95% CI: 1.013 to 1.072). This finding underscores the robustness of selenium’s effect and confirms the significance of BMI and hypertension as T2D risk factors, detailed in **Figure 6**. Additionally, sensitivity analysis using MR-pleiotropy found a P-value of 0.510, indicating no significant pleiotropic interference with the instrumental variables selected for the three exposures. This result further strengthens the reliability of our findings, suggesting that observed associations are likely specific to selenium’s effects on T2D risk and not confounded by horizontal pleiotropy.

## 4 Discussion

In this study, we employed MR to explore the potential causal relationship between 15 micronutrients and T2D. Our primary finding identified a significant positive association between selenium intake and the risk of T2D. This association persisted even after accounting for established risk factors such as BMI and hypertension in multivariate MR analyses. These results not only reinforce the understanding of selenium’s role in T2D pathogenesis but also may influence future strategies for diabetes prevention.

In both univariate and multivariate MR analyses, we identified a significant association between selenium intake and an elevated risk of T2D, which aligns partly with findings from previous studies. For instance, Demircan et al. (25) noted in their study on serum selenium and selenoprotein P levels a positive correlation with T2D among males, where a 1 mg/L increase in SELENOP corresponded to an (OR) of 1.22 (95% CI: 1.00, 1.48), particularly pronounced in male subjects, and also noted an association with hypertension, suggesting a potentially risky role of selenium in T2D etiology. Selenium, an essential micronutrient previously studied for its potential as an anticancer agent, has been associated with higher incidence of T2D in individuals using selenium for cancer prevention. Kohler et al. (26) conducted a cross-sectional analysis involving 1,727 participants from a randomized clinical trial on selenium supplementation for colorectal adenoma chemoprevention, finding that higher plasma selenium levels were significantly linked to T2D prevalence, which supports our findings. Two studies (27, 28) utilizing NHANES data to explore the association between selenium and T2D in American participants, covering over 9,000 individuals, found that higher selenium concentrations were associated with increased incidence of T2D, with ORs of 1.57 (95% CI: 1.16, 2.13) and 7.64 (95% CI: 3.34, 17.46), respectively. Stranges et al. (29) reported from the Olivetti Heart Study in Italy that participants in the highest tertile of baseline selenium levels had a higher proportion of diabetes cases compared to those in the lowest tertile. Zhang et al. (30), comparing trace element levels among Chinese participants with and without diabetes history, found that those in the highest quartile of selenium content had an OR of 2.69 (95% CI: 1.31, 3.49) compared to others. In contrast to these findings linking selenium with T2D, studies from the Nord-Trøndelag Health Study in Norway (31, 32) found no association. Simic et al. (31) reported an OR of 1.13 (95% CI: 0.65, 1.96) for diabetes prevalence in participants with the highest tertile of whole blood selenium concentration compared to the lowest, while Hansen et al. (32), focusing on early diabetes, reported an OR of 0.93 (95% CI: 0.50, 1.74). The reasons for discrepancies with Norwegian studies, where median selenium concentrations were measured within 100-105 micrograms per liter, significantly lower than mainstream studies, remain unclear. Stranges et al. (33) indicated in the Nutritional Prevention of Cancer trial that increased odds of T2D were observed specifically in individuals with blood selenium levels ≥121.6 ng/mL, suggesting a potential threshold effect of selenium levels associated with increased susceptibility to T2D that warrants further investigation in future studies. Overall, these findings suggest that higher circulating selenium concentrations or selenium supplementation for chemoprevention may moderately increase the incidence of type 2 diabetes (34).

The potential mechanistic link between selenium and T2D may be partially mediated through selenoprotein glutathione peroxidase 1 (GPx-1) (35). GPx-1, as the most abundant selenoprotein, primarily functions in the cytoplasm by converting hydrogen peroxide to water, thereby preventing lipid and protein oxidation (36–38). Mice with elevated GPx-1 expression exhibit a T2D-like phenotype characterized by obesity, hyperglycemia, hyperlipidemia, hyperinsulinemia, insulin resistance, increased pancreatic β-cell mass, glucose-stimulated insulin secretion, elevated plasma leptin levels, and hepatic lipogenesis (39). This phenotype in GPx-1-overexpressing mice can be almost completely reversed under dietary selenium deficiency or alleviated with reduced food intake by 40% (39). Prolonged GPx-1 activation in animal models may disrupt insulin signaling pathways (40, 41), and GPx-1 overexpression has been associated with obesity and insulin resistance in experimental settings (42), whereas its reduction tends to mitigate these effects (43). Moreover, liver-derived selenoprotein P (SELENOP) plays a critical role in systemic selenium homeostasis. Studies indicate that increased hepatic SELENOP mRNA expression correlates with hyperglycemia and reduced glucose tolerance in humans (44). Administration of purified SELENOP has been shown to impair insulin signaling, whereas anti-SELENOP antibodies enhance insulin signaling and glucose metabolism (45, 46). Individuals with T2D or prediabetes generally exhibit significantly higher serum SELENOP concentrations compared to those with normal glucose tolerance. Furthermore, SELENOP levels positively correlate with markers of insulin resistance, including body mass index, waist circumference, systolic blood pressure, plasma triglycerides, serum glucose, and glycated hemoglobin (47). Genetic studies involving targeted deletion and RNA interference-mediated knockdown of the Selenop gene in mice have demonstrated improved systemic insulin sensitivity and glucose tolerance, underscoring SELENOP’s potential role in regulating insulin function (45). Another potential pathway involves selenium’s essential function in the synthesis of deiodinase-2 (DIO2), a critical enzyme in thyroid hormone metabolism. Selenium status directly influences DIO2 activity, which in turn affects thyroid hormone levels. Thyroid dysfunction is linked to an increased risk of insulin resistance and T2D. Studies involving specific knockout of DIO2 in pituitary and adipose tissues in mice with normal thyroid function have shown improved glucose tolerance, insulin sensitivity, and reduced body fat (48, 49). This suggests that DIO2 activity not only impacts thyroid hormone levels but may also directly or indirectly influence glucose metabolism and the risk of T2D (50).

Another proposed mechanism relates to oxidative stress, particularly under conditions of high selenite concentrations and its metabolic product, methylselenol, in the presence of persistent hyperglycemia. Such conditions significantly elevate intracellular oxidative stress (51), leading to increased production of reactive oxygen species (ROS). If not adequately neutralized, these ROS can impair pancreatic β-cell function (52), which is particularly susceptible to oxidative damage. This impairment directly affects insulin secretion and contributes to diabetes progression (53). Selenium normally assists in neutralizing ROS through activation of the antioxidant enzyme system under physiological conditions, thereby protecting pancreatic cells from oxidative damage (54). However, excessive selenium intake can exceed this protective threshold and act as a pro-oxidant, potentially increasing oxidative stress instead of mitigating it (55). Excess selenium may interact with cellular thiol groups, disrupting protein folding and compromising cellular function. In diabetic individuals, this effect could exacerbate existing oxidative stress conditions (56).

Although selenium discovery is the focus of this study, we must not overlook the potential roles of other micronutrients in diabetes. In this study, elements such as calcium, iron, and vitamin D did not exhibit significant associations with the risk of T2D. This may be due to their weaker biological effects on diabetes risk or potential modulation by other factors. For instance, calcium may primarily relate to bone health, while both excess and deficiency of iron can impact cellular metabolism and immune function. Vitamin D’s role is more complex, influencing not only bone health but also immune system regulation and inflammatory responses. For example, in a cohort study (57) of the Spanish population, elevated serum calcium levels were associated with increased risk of developing diabetes. Researchers suggested that abnormalities in calcium homeostasis may play a significant role in the development of type 2 diabetes (HR 2.87, 95% CI 1.18–6.96, *P* = 0.02). Therefore, future research should explore the intricate effects of these elements on diabetes risk across different biological backgrounds and environmental conditions.

While our study findings support a positive correlation between selenium and the risk of T2D, we must also recognize the limitations of the MR approach. Firstly, the selection of genetic instrumental variables may be influenced by population structure, especially across different ethnicities and geographical regions. Since our sample data are derived from European populations, the generalizability of our conclusions needs validation in subsequent studies. Secondly, the biological effects of selenium may be influenced by interactions with other nutrients, which may not have been fully accounted for. Additionally, the bioactivity of selenium may depend on its chemical form and bioavailability, factors that may not have been adequately considered in our analysis.

Our study results offer new insights for future research directions. Firstly, further mechanistic studies are needed to elucidate how selenium influences the onset and progression of diabetes. Secondly, clinical trials could help validate the potential effects of selenium supplementation on the prevention and treatment of diabetes, as well as determine the optimal selenium intake. Furthermore, future research should explore interactions between selenium and other micronutrients, and consider how individual genotypes affect selenium requirements and metabolism.

## 5 Conclusion

This study systematically evaluated potential causal relationships between 15 micronutrients and the risk of T2D using MR. Multivariate analyses adjusting for confounding factors such as BMI and hypertension confirmed significant results. Our primary finding reveals a positive correlation between selenium intake and T2D risk, consistently observed across various sensitivity analyses. This supports selenium’s potential role in T2D onset and provides novel insights for future prevention and treatment strategies. These findings align with prior research linking selenium to T2D risk, while highlighting the complex nature of interpreting such associations, including selenium’s biological mechanisms and interactions with other micronutrients. Although other micronutrients did not show significant associations with T2D risk in this study, our research underscores the importance of comprehensively considering micronutrients in T2D research. In summary, this study advances understanding of selenium’s role in T2D development and suggests promising avenues for further research and clinical applications. These findings warrant validation in broader biological contexts.

## Data Availability

The original contributions presented in the study are included in the article/**Supplementary Material**. Further inquiries can be directed to the corresponding author.
