# Supplementary material for: Tracing links between micronutrients and type 2 diabetes risk: the singular role of selenium
Source: Front Endocrinol (Lausanne). 2024 Oct 14;15:1422796. doi: 10.3389/fendo.2024.1422796 (PMC11513319; doi:10.3389/fendo.2024.1422796)
Supplement: Supplementary file 1 [file Table1.docx]

**STROBE-MR checklist of recommended items to address in reports of Mendelian randomization studies**^1^ ^2^

| **Item No.** | **Section** | **Checklist item** | **Page No.** | **Relevant text from manuscript** |
| --- | --- | --- | --- | --- |
| 1 | **TITLE and ABSTRACT** | Indicate Mendelian randomization (MR) as the study’s design in the title and/or the abstract if that is a main purpose of the study | Abstract | This study aimed to evaluate the potential causal relationships between 15 major micronutrients and T2D risk using both univariate and multivariate Mendelian randomization methods and to investigate the specific roles of significant micronutrients in T2D. |
|  | **INTRODUCTION** |  |  |  |
| 2 | **Background** | Explain the scientific background and rationale for the reported study. What is the exposure? Is a potential causal relationship between exposure and outcome plausible? Justify why MR is a helpful method to address the study question | Background | While numerous studies have disclosed the genetic foundations of T2D, environmental factors—particularly micronutrient intake—seem to play an equally pivotal role in the disease's onset. Micronutrients like calcium, magnesium, iron, and zinc are crucial not only for maintaining metabolic health but also for regulating glucose metabolism through their influence on insulin secretion and activity. For instance, magnesium serves as a cofactor for over 300 enzymes, essential for glucose metabolism and insulin signaling. Despite extensive exploration in epidemiological studies, the link between micronutrients and T2D remains ambiguous, marred by potential confounders and reverse causation. |
| 3 |  | State specific objectives clearly, including pre-specified causal hypotheses (if any). State that MR is a method that, under specific assumptions, intends to estimate causal effects | Materials and Methods | This study employed MR to investigate the potential causal relationships between fifteen micronutrients and T2D. Initially, genetic instrumental variables (single nucleotide polymorphisms, SNPs) associated with these micronutrients were extracted from published GWAS. These instrumental variables were then utilized to conduct preliminary two-sample MR analyses to screen for micronutrients showing significant associations with T2D risk. Building on this initial analysis, we incorporated two well-known risk factors—body mass index (BMI) and hypertension—that are strongly correlated with T2D. This inclusion allowed us to perform multivariate MR analyses to ascertain the independent associations between selenium and other confounders with T2D risk. |
|  | **METHODS** |  |  |  |
| 4 | **Study design and data sources** | Present key elements of the study design early in the article. Consider including a table listing sources of data for all phases of the study. For each data source contributing to the analysis, describe the following: | Materials and Methods | The GWAS data for the 15 trace elements in our study data source were obtained from IEU OpenGWAS, with specific ID numbers shown in Supplementary Table 1, while the GWAS data for T2D were obtained from the latest R10 version of the Finnish Genetic Data Bank, with the ID number finngen_R10_T2D. |
|  | a) | Setting: Describe the study design and the underlying population, if possible. Describe the setting, locations, and relevant dates, including periods of recruitment, exposure, follow-up, and data collection, when available. | Materials and Methods | Information about the ieu database and the FinnGen biobank is described in the data sources, including when the samples were collected (see Supplementary Table 1 for details), the study site (Europe), and the study population (European origin). |
|  | b) | Participants: Give the eligibility criteria, and the sources and methods of selection of participants. Report the sample size, and whether any power or sample size calculations were carried out prior to the main analysis | Materials and Methods | This study reports the sample sizes of study subjects from the IEU database and the FinnGen biospecimen library, with exposure data of 64,979 and 2,603 cases, respectively. Outcome data included 65,085 patients with type 2 diabetes and 335,112 controls. Sample size calculations were not reported. |
|  | c) | Describe measurement, quality control and selection of genetic variants | Materials and Methods | Instrumental variables in the study were obtained from the IEU database and the FinnGen biobank, and the screening process and criteria for the genetic variant instrumental variables are also reported in the article, as described in the methodology section of the article |
|  | d) | For each exposure, outcome, and other relevant variables, describe methods of assessment and diagnostic criteria for diseases | Materials and Methods | This study describes the definition of the outcome in the selection of genetic instrumental variables for methodology and provides the references on which the definition is based. The definitions of fibromyalgia refer to the International Classification of Diseases, 8th, 9th and 10th editions, respectively. |
|  | e) | Provide details of ethics committee approval and participant informed consent, if relevant | Materials and Methods | Ethical approval and informed consent of the patients were obtained during the collection of gaws data for this study and the current MR was also approved by the Swedish Ethical Review Board, approval number: 2019-02793 |
| 5 | **Assumptions** | Explicitly state the three core IV assumptions for the main analysis (relevance, independence and exclusion restriction) as well assumptions for any additional or sensitivity analysis | Background | This paper provides a preliminary description of the three core hypotheses, but the core hypotheses are reflected in the analytical methods. In addition, three methods for sensitivity analyses and horizontal polytropy detection are described: the weighted median method, MR-Egger, and MR-PRESSO, and the use of the F-statistic to calculate statistical validity and the Q-statistic to detect heterogeneity is reported |
| 6 | **Statistical methods: main analysis** | Describe statistical methods and statistics used |  |  |
|  | a) | Describe how quantitative variables were handled in the analyses (i.e., scale, units, model) | Materials and Methods | Studies reported analyses using random-effects models when there were >3 instrumental variables for genetic variants, otherwise fixed-effects models were used; statistical effect sizes or units of measurement for exposure, outcome, and relevant covariates were not transformed and therefore not reported |
|  | b) | Describe how genetic variants were handled in the analyses and, if applicable, how their weights were selected | Materials and Methods | A stringent criterion was employed to identify instrumental variables for substituting the exposure, involving the restriction of the p-value and exclusion of linkage disequilibrium (P < 5 × 10-6, R2<0.001, kb = 10000). This method was widely used in previous MR studies. 16-17 Furthermore, the strength of each selected SNP was evaluated by calculating the F-statistic using the formula.18 F = [(N - k - 1) / k]×[R² / (1 - R²)] |
|  | c) | Describe the MR estimator (e.g. two-stage least squares, Wald ratio) and related statistics. Detail the included covariates and, in case of two-sample MR, whether the same covariate set was used for adjustment in the two samples | Materials and Methods | The genetic tools used in this paper to report exposure were derived from the results of a GWAS, and the study population consisted of two groups of 64,979 and 2,603 adults of European ancestry, respectively, with no analyses adjusted for age, sex, or study site using genetic modelling. In addition, the specific MR statistical methods and software used are described in detail in the text |
|  | d) | Explain how missing data were addressed | - | No lost data was found |
|  | e) | If applicable, indicate how multiple testing was addressed | Materials and Methods | Fifteen micronutrients were reported in the study, and although only selenium was significant in the simple two-sample analysis, the results were still significant when hypertension and BMI were added to the multivariate magnetic resonance analyses to exclude possible confounding factors |
| 7 | **Assessment of assumptions** | Describe any methods or prior knowledge used to assess the assumptions or justify their validity | Materials and Methods | The F-statistic was used to estimate statistical power in this study and no other method was used |
| 8 | **Sensitivity analyses and additional analyses** | Describe any sensitivity analyses or additional analyses performed (e.g. comparison of effect estimates from different approaches, independent replication, bias analytic techniques, validation of instruments, simulations) | Materials and Methods | Cochran’s Q-test for IVW and MR-Egger was employed to detect potential violations of the assumption by assessing the heterogeneity of the association between individual IVs. The IVs included in the analysis were considered non-heterogeneous when the p-value exceeded 0.05. In cases where no significant heterogeneity was observed (p>0.05), the default fixed-effects model was utilized. Conversely, the random-effects model was employed if substantial heterogeneity was present (p<0.05). MR-Egger was applied to estimate horizontal pleiotropy based on its intercept, ensuring that genetic variation was independently associated. |
| 9 | **Software and pre-registration** |  |  |  |
|  | a) | Name statistical software and package(s), including version and settings used | Materials and Methods | TwoSampleMR package (version 0.5.6) in R 4.3.2 |
|  | b) | State whether the study protocol and details were pre-registered (as well as when and where) | - | Unfortunately, the study protocol and details were not pre-registered. At the initiation of the study, we did not initially consider or recognize the necessity of pre-registration. We appreciate your understanding of this limitation. |
|  | **RESULTS** |  |  |  |
| 10 | **Descriptive data** |  |  |  |
|  | a) | Report the numbers of individuals at each stage of included studies and reasons for exclusion. Consider use of a flow diagram | Materials and Methods | The example text provides the number of sample populations in the methodology section and does not provide information about the populations in the results. Information on excluded populations with reasons for exclusion is not provided and the flow chart is shown in Figure 1 |
|  | b) | Report summary statistics for phenotypic exposure(s), outcome(s), and other relevant variables (e.g. means, SDs, proportions) | - | The example text provides information in Annex Table 1, which presents details of the characteristics of the study population, exposures. Information on potential confounders is provided in Supplementary Table 3. |
|  | c) | If the data sources include meta-analyses of previous studies, provide the assessments of heterogeneity across these studies |  | No data from meta-analysis found |
|  | d) | For two-sample MR:  i.  Provide justification of the similarity of the genetic variant-exposure associations between the exposure and outcome samples  ii.  Provide information on the number of individuals who overlap between the exposure and outcome studies | - | This study is a two-sample and multivariate MR, and in Annex Table 1 all sample populations are listed as being from Europe, so there is very little ethnographic heterogeneity, and adjustments were made for age, gender, and other factors. The study did not report information on overlapping populations. |
| 11 | **Main results** |  |  |  |
|  | a) | Report the associations between genetic variant and exposure, and between genetic variant and outcome, preferably on an interpretable scale | - | This study reports the content of this entry in Tables 1 of the Annex, including the number of instrumental variable SNPs, sample size, adjusted confounders, correlation between exposure-instrumental variables, and statistical validity. |
|  | b) | Report MR estimates of the relationship between exposure and outcome, and the measures of uncertainty from the MR analysis, on an interpretable scale, such as odds ratio or relative risk per SD difference | Results | Much of the space for the results of this study was devoted to reporting on the content of the entries, e.g. the OR for the association of selenium with type 2 diabetes mellitus was 1.040 with a 95% CI of (1.009, 1.082) |
|  | c) | If relevant, consider translating estimates of relative risk into absolute risk for a meaningful time period | Results | For example, selenium has been found to be a risk factor for type 2 diabetes, with a 4 per cent increase in the risk of fibromyalgia for every 1 unit increase in selenium levels. |
|  | d) | Consider plots to visualize results (e.g. forest plot, scatterplot of associations between genetic variants and outcome versus between genetic variants and exposure) | Results | Figure 4 |
| 12 | **Assessment of assumptions** |  |  |  |
|  | a) | Report the assessment of the validity of the assumptions | Results | The results of the assessment of the validity of the hypotheses of interest are reported in several places throughout the study, as in Table 4.For example, in Annex Table 4, the statistical validity of the instrumental variables in the associations is reported, and the results are expressed in terms of the values of the F-statistic. The heterogeneity of the statistical models was tested in the methodology using the Q-statistic to assess their stability. |
|  | b) | Report any additional statistics (e.g., assessments of heterogeneity across genetic variants, such as *I^2^*, Q statistic or E-value) | Results | The example text reports the results of the Q statistic in the results, demonstrating its significance. |
| 13 | **Sensitivity analyses and additional analyses** |  |  |  |
|  | a) | Report any sensitivity analyses to assess the robustness of the main results to violations of the assumptions | Results | The example article calculates the results of MR analyses for five, but only presents the results of MR analyses obtained by two analytical methods, including the weighted median method and IVW, which are shown in Figures 2 and 4. In addition, sensitivity analyses, including the MR egger and the MR Pleiotropy methods, are shown in the article. |
|  | b) | Report results from other sensitivity analyses or additional analyses | Results | The example text reports on the results of other analyses, such as the leave-one-out method, in Figures 4. |
|  | c) | Report any assessment of direction of causal relationship (e.g., bidirectional MR) | Results | This study has controlled for confounding under multivariate Mendelian randomisation analyses and did not report reverse results. |
|  | d) | When relevant, report and compare with estimates from non-MR analyses | Results | Non-MR results are not reported in the Results section of this paper at this time, but are discussed in the Discussion section . |
|  | e) | Consider additional plots to visualize results (e.g., leave-one-out analyses) | Results | Figure 4 |
|  | **DISCUSSION** |  |  |  |
| 14 | **Key results** | Summarize key results with reference to study objectives | Discussion | The example text reports all the key findings in the first paragraph of the discussion: selenium is a risk factor for the development of type 2 diabetes and there is a positive correlation between the two. |
| 15 | **Limitations** | Discuss limitations of the study, taking into account the validity of the IV assumptions, other sources of potential bias, and imprecision. Discuss both direction and magnitude of any potential bias and any efforts to address them | Discussion | The limitations of the study are discussed at great length in the example essay in terms of the data sources, the three main hypotheses and the analytical process in a detailed manner. |
| 16 | **Interpretation** |  |  |  |
|  | a) | Meaning: Give a cautious overall interpretation of results in the context of their limitations and in comparison with other studies | Discussion | This entry is discussed at maximum length in the example paper, and the MR results are rationalised by comparison with several published studies. |
|  | b) | Mechanism: Discuss underlying biological mechanisms that could drive a potential causal relationship between the investigated exposure and the outcome, and whether the gene-environment equivalence assumption is reasonable. Use causal language carefully, clarifying that IV estimates may provide causal effects only under certain assumptions | Discussion | Examples describe detailed biological mechanisms, such as selenium's ability to cause increased oxidative stress, which stimulates the onset and development of type 2 diabetes. |
|  | c) | Clinical relevance: Discuss whether the results have clinical or public policy relevance, and to what extent they inform effect sizes of possible interventions | Discussion | The instrumental variable SNP in this paper may be subject to a number of other possible confounding influences, and there may also be interactions between genetic and environmental factors, so the MR results are also subject to uncertainty, but conclusions can still be drawn. |
| 17 | **Generalizability** | Discuss the generalizability of the study results (a) to other populations, (b) across other exposure periods/timings, and (c) across other levels of exposure | Discussion | The example text reports in the Discussion that "In addition, the restriction of this study to populations of European origin, while reducing the potential for demographic bias, limits the generalisability of the MR results to other populations". |
|  | **OTHER INFORMATION** |  |  |  |
| 18 | **Funding** | Describe sources of funding and the role of funders in the present study and, if applicable, sources of funding for the databases and original study or studies on which the present study is based | Funding | The authors declare that financial support was received for the research, authorship, and/or publication of this article. The authors disclose that they have no business or financial associations that could create potential conflicts of interest. This study was supported by the "Academic Reserve Talent Cultivation Programme for Double First-class High-level Universities" and the "National TCM Expert Workshop Construction Project" of Guangzhou University of Traditional Chinese Medicine (Huang Feng Workshop N75, 2022).'' |
| 19 | **Data and data sharing** | Provide the data used to perform all analyses or report where and how the data can be accessed, and reference these sources in the article. Provide the statistical code needed to reproduce the results in the article, or report whether the code is publicly accessible and if so, where | Data and data sharing | The GWAS data for the 15 micronutrients were obtained from the IEU OpenGWAS database, with the specific ID numbers shown in Supplementary Table 1. In addition, the GWAS data for type 2 diabetes were obtained from the latest R10 version of the Finnish Genetic Data Bank, with the ID number finngen_R10_T2D. |
| 20 | **Conflicts of Interest** | All authors should declare all potential conflicts of interest | Conflicts of Interest | The authors declare that this study was conducted without any commercial or financial relationship, which could be interpreted as a potential conflict of interest. |

This checklist is copyrighted by the Equator Network under the Creative Commons Attribution 3.0 Unported (CC BY 3.0) license.

1. Skrivankova VW, Richmond RC, Woolf BAR, Yarmolinsky J, Davies NM, Swanson SA, et al. Strengthening the Reporting of Observational Studies in Epidemiology using Mendelian Randomization (STROBE-MR) Statement. JAMA. 2021;under review.

2. Skrivankova VW, Richmond RC, Woolf BAR, Davies NM, Swanson SA, VanderWeele TJ, et al. Strengthening the Reporting of Observational Studies in Epidemiology using Mendelian Randomisation (STROBE-MR): Explanation and Elaboration. BMJ. 2021;375:n2233.
